# Expression of inducible nitric oxide synthase in healthy pleura and in malignant mesothelioma

**DOI:** 10.1054/bjoc.2000.1384

**Published:** 2000-09-04

**Authors:** Y Soini, K Kahlos, A Puhakka, E Lakari, M Säily, P Pääkkö, V Kinnula

**Affiliations:** Departments of Pathology and Internal Medicine, University of Oulu and Oulu University Hospital, Oulu, Finland

**Keywords:** mesothelioma, NOS, pleura, apoptosis, angiogenesis

## Abstract

In this study we investigated the immunohistochemical expression of inducible nitric oxide synthase (iNOS) in a set of normal pleural mesothelial tissues, malignant mesotheliomas, mesothelioma cell lines and metastatic pleural adenocarcinomas. Furthermore, the expression of mRNA was assessed in four malignant mesothelioma cell lines in culture. Apoptosis and vascular density in malignant mesotheliomas was assessed by the TUNEL method and by immunohistochemistry with an antibody against FVIII-related antigen. Immunohistochemically mesothelial cells in non-neoplastic healthy pleural tissues were mostly negative for iNOS. Positivity for iNOS was observed in 28/38 (74%) and 24/25 (96%) of malignant mesotheliomas and metastatic pleural adenocarcinomas, respectively. Epithelial and mixed mesotheliomas expressed more often strong iNOS immunoreactivity compared to the sarcomatoid subtype (*P* = 0.023). Moreover, metastatic adenocarcinomas expressed more often iNOS positivity than mesotheliomas (*P* = 0.021). Experiments with the cell lines confirmed that malignant mesothelioma cells are capable of synthesizing iNOS. No significant association was found between iNOS expression and apoptosis or vascular density in malignant mesotheliomas. The higher expression of iNOS in the epithelial subtype of mesothelioma and pleural metastatic adenocarcinoma might be due to an increased sensitivity of these cell types to cytokine-mediated iNOS upregulation. The strong expression of iNOS suggests a putative role for NO in the growth and progression of these tumours. © 2000 Cancer Research Campaign


*British Journal of Cancer* (2000) **83**(7), 880–886
© 2000 Cancer Research Campaign
doi: 10.1054/ bjoc.2000.1384, available online at http://www.idealibrary.com on IDEAL®

### Expression of inducible nitric oxide synthase in healthy pleura and in malignant mesothelioma

**Y Soini, K Kahlos, A Puhakka, E Lakari, M Säily, P Pääkkö and V Kinnula**

Departments of Pathology and Internal Medicine, University of Oulu and Oulu University Hospital, Oulu, Finland

**Summary** In this study we investigated the immunohistochemical expression of inducible nitric oxide synthase (iNOS) in a set of normal pleural mesothelial tissues, malignant mesotheliomas, mesothelioma cell lines and metastatic pleural adenocarcinomas. Furthermore, the expression of mRNA was assessed in four malignant mesothelioma cell lines in culture. Apoptosis and vascular density in malignant mesotheliomas was assessed by the TUNEL method and by immunohistochemistry with an antibody against FVIII-related antigen. Immunohistochemically mesothelial cells in non-neoplastic healthy pleural tissues were mostly negative for iNOS. Positivity for iNOS was observed in 28/38 (74%) and 24/25 (96%) of malignant mesotheliomas and metastatic pleural adenocarcinomas, respectively. Epithelial and mixed mesotheliomas expressed more often strong iNOS immunoreactivity compared to the sarcomatoid subtype ($P = 0.023$). Moreover, metastatic adenocarcinomas expressed more often iNOS positivity than mesotheliomas ($P = 0.021$). Experiments with the cell lines confirmed that malignant mesothelioma cells are capable of synthesizing iNOS. No significant association was found between iNOS expression and apoptosis or vascular density in malignant mesotheliomas. The higher expression of iNOS in the epithelial subtype of mesothelioma and pleural metastatic adenocarcinoma might be due to an increased sensitivity of these cell types to cytokine-mediated iNOS upregulation. The strong expression of iNOS suggests a putative role for NO in the growth and progression of these tumours. © 2000 Cancer Research Campaign

**Keywords:** mesothelioma; NOS; pleura; apoptosis; angiogenesis

Malignant mesothelioma is a tumour with a poor prognosis (Walz and Koch, 1985). The development of mesothelioma is associated with an occupational exposure to asbestos fibres in most patients (Mossman et al, 1996). The pathogenesis of malignant mesothelioma is still far from clear but there is considerable evidence that reactive oxygen and nitrogen species (ROS and RNS, respectively) play an important role in the pathogenesis of this disease (Kamp and Weitzman, 1999). Asbestos fibres increase the generation of RNS and the levels of nitrotyrosine, a marker of NO related cell damage, at least in rat pleural mesothelium (Tanaka et al, 1998; Kamp and Weitzmann, 1999; Kinnula, 1999). It can therefore be hypothesized that the expression of nitric oxide synthases might be upregulated in asbestos-related pleural diseases.

NO is generated from L-arginine by nitric oxide synthase (NOS) which is present in three isoforms, endothelial NOS (eNOS), neuronal NOS (nNOS) and inducible NOS (iNOS) (Kobzik et al, 1993; Beckman and Koppenol, 1996; Wink and Mitchell, 1998). The synthesis of the constitutive forms of NOS, eNOS and nNOS, is dependent on the calcium concentration of the cell. Inducible NOS is stimulated by several cytokines, such as IL-1, IL-4, IL-10, IL-12, INF-γ, TNF-α and β and by asbestos fibres in a variety of cells (Thomas et al, 1994; Quinlan et al, 1998; Tanaka et al, 1998; Kamp and Weitzmann, 1999; Kinnula, 1999). Nitric oxide is a free radical, which reacts with molecules, such as superoxide, thiols and metalloproteins thus locally modifying biological reactions of the cell (Beckman and Koppenol, 1996; Wink and Mitchell, 1998). One of the most potential toxic compounds generated in these reactions is peroxynitrite, which can contribute to the formation of other reactive nitrogen metabolites in the cell.

Inducible NOS can be detected in multiple cells such as macrophages, bronchial epithelial cells, alveolar type II epithelial cells, fibroblasts and pleural mesothelial cells (Kobzik et al, 1993; Thomas et al, 1994; Moilanen et al, 1997; Quinlan et al, 1998; Tanaka et al, 1998). The NOS family of proteins also plays an important role in many pathophysiological conditions. The expression of iNOS is increased in inflammatory diseases, such as asthma (Belvisi et al, 1995; Li, 1997) and interstitial lung diseases (Saleh et al, 1997). Nitrotyrosine is a marker of NO-mediated cell injury, and it is accumulated in idiopathic pulmonary fibrosis (Saleh et al, 1997) and at least in pleural mesothelium of asbestos exposed rats (Choe et al, 1998; Tanaka et al, 1998).

Nitric oxide production may also lead to DNA damage and thus promote carcinogenesis (Ambs et al, 1997; Kamp and Weitzman, 1999). This is especially interesting with asbestos fibres since they have been shown to cause NO generation, induction of iNOS and contribute to mesothelial cell damage by reactive nitrogen species (Choe et al, 1998; Tanaka et al, 1998). Asbestos fibres are genotoxic, they cause formation of 8-hydroxyguanosine, a marker of DNA damage. This effect has been shown to be attenuated by NAME, a specific inhibitor of NOS (Chao et al, 1996; Chen et al, 1996). Inducible NOS has also been shown to be expressed in many tumours such as breast, lung, prostate, gynaecological and colon carcinomas and in B-cell chronic lymphocytic leukemias (Thomsen et al, 1994; Thomsen et al, 1995; Ambs et al, 1998a; Ambs et al, 1998b; Klotz et al, 1998; Zhao et al, 1998). We are not

*Received 14 January 2000*
*Revised 28 April 2000*
*Accepted 8 June 2000*

*Correspondence to:* Y Soini aware of any studies on iNOS or other forms of nitric oxide synthases in human healthy pleura or malignant mesothelioma.

Given the suggested role of NO in asbestos induced pleural disorders, carcinogenesis and tumour progression, we investigated the expression of iNOS in normal healthy and reactive pleural mesothelium and malignant pleural mesothelioma. Five pleural samples containing non-inflamed and non-neoplastic mesothelium, four cases containing inflamed reactive mesothelium and 38 cases of malignant mesotheliomas were stained with two antibodies to iNOS. Furthermore, 25 metastatic pleural adenocarcinomas were included in the study material to test whether any differences could be found in the iNOS immunoreactivity between such tumours and mesotheliomas with different progenitor cell type, pathogenesis and tumour growth characteristics. Since NO has been suggested to influence apoptosis and angiogenesis we also determined the extent of apoptosis in mesotheliomas by TUNEL and also immunostained the slides with an antibody to FVIII. The mRNA and protein expression of iNOS was further assessed in four malignant mesothelioma cell lines (M14K, M25K, M28K and M38K) and a nonmalignant human transformed mesothelial cell line (Met5A) in culture.

### MATERIALS AND METHODS

### Histological material

Altogether 38 malignant mesotheliomas, 25 pleural metastatic adenocarcinomas, four cases of inflamed mesothelium and five cases originating from peripheral lung tissue containing non-neoplastic and non-inflamed pleura were retrieved from the files of the Department of Pathology, Oulu University Hospital between 1976–1997. All the material had been fixed in 10% buffered formalin and embedded in paraffin. Malignant mesotheliomas were subclassified into epithelial, sarcomatoid and biphasic subtypes according to the criteria given by AFIP (Battifora and McCaughet, 1994). Metastatic adenocarcinomas consisted of tumours originating from lung, breast, kidney and liver. Clinical data such as the sex, age and survival of the patients was obtained from the hospital records. In the mesothelioma group the mean age was 62.2 ± 10.4 years. There were 5 women and 33 men. The mean survival in the mesothelioma group was 15.7 ± 27.3 months.

### Immunohistochemical stainings for iNOS

A rabbit polyclonal antibody against iNOS (sc-651) was purchased from Santa Cruz Biotechnology (Santa Cruz Biotechnology Inc, Santa Cruz, CA) and a mouse monoclonal anti-iNOS antibody, (N-32320) from Transduction Laboratories (Transduction Laboratories, Lexington, KY). According to the manufacturers, both antibodies recognize mouse, rat and human iNOS.

The immunostainings with the poly- and monoclonal iNOS antibodies were performed as follows. Before application of the primary antibodies, the sections were heated in a microwave oven in 10 mM citric acid monohydrate, pH 6.0, for 10 minutes. The dilution for the primary antibody for the poly- and monoclonal iNOS antibody was 1:200 and 1:60, respectively. The immunostaining was performed using the Histostain-Plus Bulk Kit (Zymed Laboratories Inc, South San Francisco, CA) and the chromogen used was aminoethyl carbazole (AEC) (Zymed Laboratories Inc). Negative control stainings were carried out by substituting PBS or non-immune mouse or rabbit serum for the primary antibodies.

The intensity of the immunostainings with all the antibodies was evaluated by dividing the staining reaction in four groups.

1 = weak cytoplasmic staining intensity, corresponding approximately to less than 25% of the staining intensity in neutrophils;
2 = moderate cytoplasmic staining intensity, corresponding approximately to 25–50% of the staining intensity in neutrophils;
3 = strong cytoplasmic staining intensity, corresponding approximately to 50–75% of the staining intensity in neutrophils;
4 = very strong cytoplasmic staining intensity, corresponding approximately to 75% or more of the staining intensity in neutrophils.

The quantity of the immunostaining was evaluated as follows:
0 = no positive immunostaining;
1 = < 25% of tumour cells showing cytoplasmic positivity;
2 = 25–50% of tumour cells showing cytoplasmic positivity;
3 = 50–75% of tumour cells showing cytoplasmic positivity;
4 = > 75% of tumour cells showing cytoplasmic positivity.

A combined score for the immunostaining, based on both qualitative and quantitative immunostaining was composed by adding both the qualitative and quantitative score which was then divided in three main groups; – = no immunostaining (score 0); + = weak immunostaining (scores 1–4); ++ = strong immunostaining (scores 5–8).

### Immunostaining of FVIII related antigen

A polyclonal rabbit anti-human antibody to FVIII-related antigen was purchased from Dako (Dakopatts, Denmark). The primary antibody was applied on the slides for 1 hour with a dilution of 1:50. After this a secondary anti-rabbit antibody (Dako, Dakopatts, Denmark) was applied followed by the ABC-complex. The colour was developed with diaminobenzidine and hydrogen peroxide. The vascular density was estimated as the number of positively stained blood vessels in one high power field. In each tumour section a minimum of 10 high power fields were analysed.

### 3′-end labelling of DNA in apoptotic cells

In order to detect apoptotic cells, in situ labelling of the 3′-ends of the DNA fragments generated by apoptosis-associated endonucleases was performed using the ApopTag in situ apoptosis detection kit (Oncor, Gaithersburg, MD, USA). The sections, after being dewaxed in xylene and rehydrated in ethanol, were incubated with 20 μg/ml Proteinase K (Boehringer Mannheim GmbH, Mannheim, Germany) at room temperature for 15 minutes. The endogenous peroxidase activity was blocked by incubating the slides in 2% hydrogen peroxide in PBS, pH 7.2. The slides were then treated with terminal transferase enzyme and digoxigenin-labelled nucleotides after which anti-digoxigenin-peroxidase solution was applied on the slides. The colour was developed with diaminobenzidine after which the slides were lightly counterstained with haematoxylin. Cells were defined as apoptotic if the whole nuclear area of the cell labelled positively. Apoptotic bodies were defined as small positively-labelled globular bodies in the cytoplasm of the tumour cells which could be found either singly

© 2000 Cancer Research Campaign

**Table 1** Immunohistochemical expression of iNOS in non-neoplastic mesothelium, mesotheliomas and metastatic adenocarcinomas studied with the poly- and monoclonal iNOS antibody.

| INOS Polyclonal | Non-neoplastic mesothelium | Reactive mesothelium | Epithelial mesothelioma | Sarcomatoid mesothelioma | Biphasic mesothelioma | Metastatic adenocarcinoma |
|---|---|---|---|---|---|---|
| – | 4 | 0 | 2 | 6 | 2 | 1 |
| + | 1 | 3 | 17 | 4 | 2 | 18 |
| ++ | 0 | 1 | 5 | 0 | 0 | 6 |
| Total | 5 | 4 | 24 | 10 | 4 | 25 |
| **INOS Monoclonal*** | | | | | | |
| – | 4 | 0 | 6 | 7 | 2 | 3 |
| + | 1 | 3 | 12 | 2 | 2 | 21 |
| ++ | 0 | 1 | 3 | 0 | 0 | 1 |
| Total | 5 | 4 | 21 | 9 | 4 | 25 |

*= Due to exhaustion of the blocks immunostaining of some cases with the monoclonal antibody are missing or in groups. To estimate the apoptotic index (the percentage of apoptotic events in a given area), apoptotic cells and bodies in tumour cells were counted in 10 high power fields (HPFs) and this figure was divided by the number of tumour cells in the same HPFs.

### Cultured cells

Mesothelioma cell lines M14K, M25K, M28K and M38K were originally established from the tumour tissue of untreated patients (Pelin-Enlund et al, 1990). Human non-malignant transformed pleural mesothelial cells (Met5A) were obtained from American Type Culture Collection (Rockville, MD). The cells were grown in RPMI 1640 cell culture medium supplemented with 10% fetal calf serum, 100 U/ml penicillin, 100 µg/ml streptomycin, and 0.03% L-glutamine (all from LTI, Life Technologies, Paisley, UK) at 37°C in 5% $CO_2$ atmosphere. For immunocytochemistry the cell pellets were fixed in 10% neutral formalin overnight, after which the formalin was removed, and melted 2% agar was laid over the pellets. The agar blocks were further embedded in paraffin. Four µm thick sections were cut from the cell blocks and stained for the polyclonal anti-iNOS antibody as previously described.

### RT-PCR for iNOS

Expression of iNOS mRNA in cultured cells was investigated by using reverse transcription polymerase chain reaction (RT-PCR). The oligonucleotide primers were selected according to cDNA sequence data published earlier (Geller et al, 1993). Total cellular RNA was extracted from the cells using a kit for RNA isolation (RNEasy, Qiagen, Hilden, Germany). One µg of RNA was treated with DNAase I (Pharmacia, Biotech, Milwaukee, WI) at 37°C for 10 min and at 75°C for 10 min to eliminate possible DNA contamination of the samples and reverse transcribed with 100 U of Moloney murine leukaemia virus reverse transcriptase (Gibco BRL, Paisley, UK) and 5 pmol of antisense primer (5′-GGTGCT-GCTTGTTAGGAGGTCAAGTAAAGGGC-3′) at 42°C for 45 min in a 20 µl reaction mixture containing 1 U RNase inhibitor (5 Prime – >3 Prime, Boulder, CO). The cDNA was PCR-amplified in a thermal cycler (Perkin-Elmer Cetus, Norwalk, CT) using 1 U of DNA polymerase (Dynazyme, Finnzymes, Finland) and 5 pmol of sense primer (5′-TGCCTGGCAAGCCCAAGGTC-TATGTTCAGGAC-3′) in a 100 µl reaction volume containing 1.5 mM $MgCl_2$. The thermal profile involved 35 cycles of denaturation at 94°C for 50 sec, primer annealing at 64°C for 50 sec, and extension at 72°C for 1 min 30 sec. PCR products were electrophoresed in an ethidium bromide-stained 2% agarose (Seakem, Rockland, ME) gel and visualised under UV-light. The amplification product was 500 base pairs (bp) in length. Negative controls were established in each experiment by substituting the RNA sample with water and by leaving the reverse transcriptase enzyme out of the RT-reaction. A549 cells were used as positive controls in each experiment (Zhao et al, 1998).

### Statistical analysis

SPSS for Windows (Chicago, IL, USA) was used for statistical analysis. The significance of the associations were determined using Fisher's exact probability test, correlation analysis and two-tailed *t*-test. The survival analysis was performed by the Kaplan-Meyer curve and significance of associations were tested by the log-rank test. Probability values $P \leq 0.05$ were considered statistically significant.

### RESULTS

Mesothelial cells in four out of five cases in non-neoplastic and non-inflamed healthy pleural tissue showed no immunoreactivity for iNOS (Fig. 1A) (Table 1). In one case a few, scattered iNOS positive mesothelial cells could be observed amongst a majority of negatively stained cells (not shown). In contrast, all cases with inflamed reactive mesothelium showed iNOS positivity and in one case, the expression was strong. Malignant pleural tissues also showed positive immunostaining for iNOS in most of the cases; 28/38 (74%) of malignant pleural mesotheliomas (Fig. 1B, C) and 24/25 (96%) of metastatic pleural adenocarcinomas were iNOS positive (Fig. 1 D, E) (Table 1). The immunoreactivity for iNOS was diffuse intracytoplasmic and finely granular.

Of the non-neoplastic cells, strong immunoreactivity for iNOS could be observed in lung alveolar macrophages and in macrophages of the tumour tissue. Also neutrophils expressed strong positivity for iNOS while lymphocytes were negative.

 *© 2000 Cancer Research Campaign*

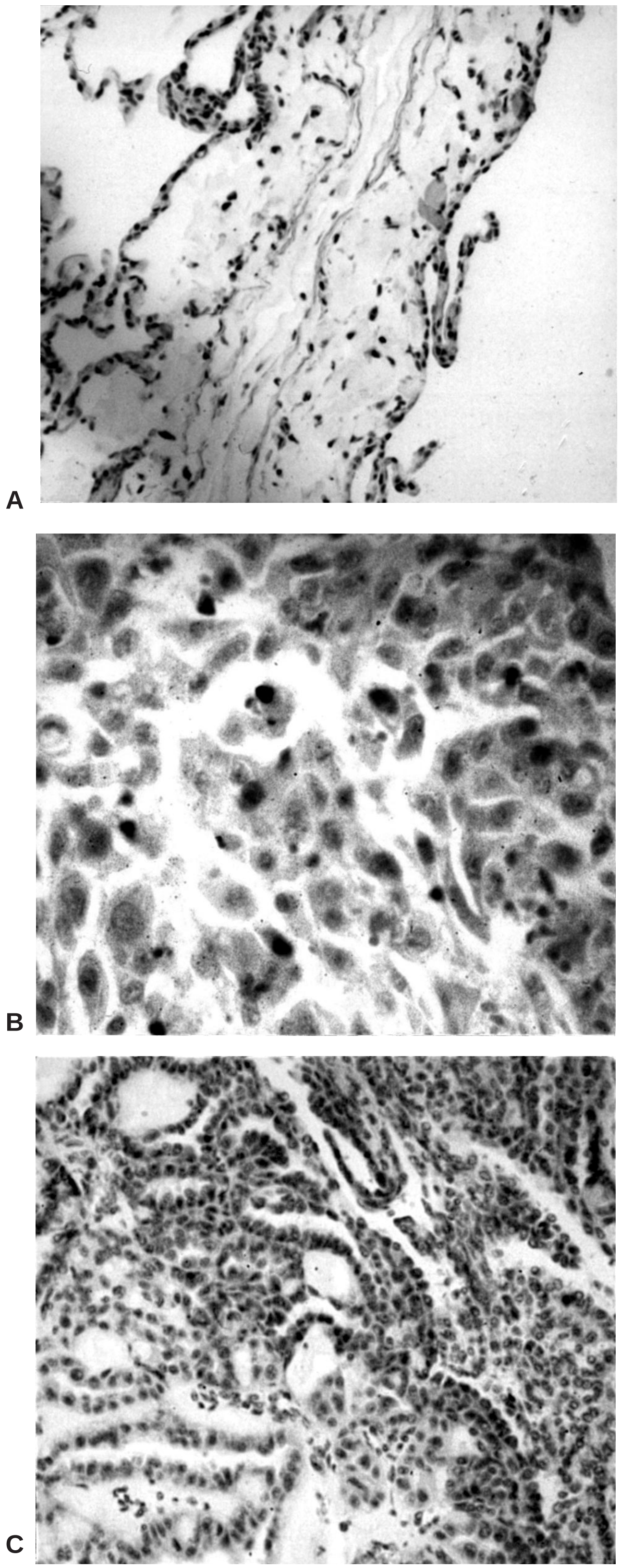

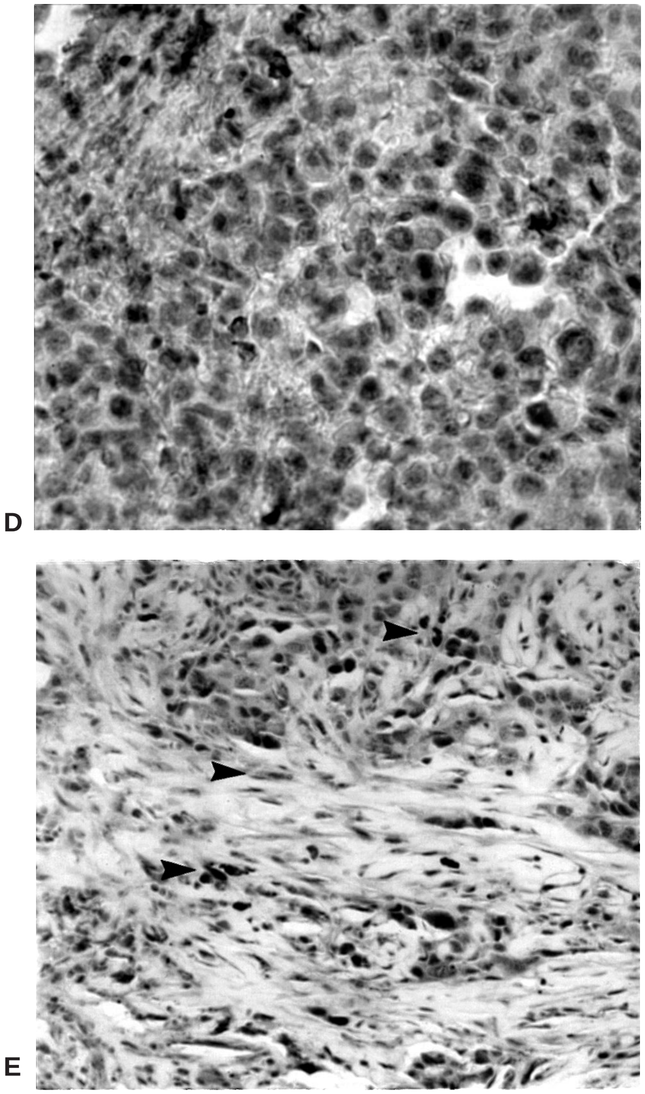

**Figure 1** **A.** Immunostaining for iNOS in normal pleural samples. Mesothelial cells lining the healthy pleura tissue are negative for iNOS. Diffusely scattered macrophages seen in the subpleural tissue are, however, positive. **B.** Globular cytoplasmic immunoreactivity for iNOS can be seen in tumour cells of an epithelial type of malignant mesothelioma with the polyclonal iNOS antibody **C.** Another epithelial mesothelioma case stained with the monoclonal iNOS antibody. The tumour cells show diffuse cytoplasmic positivity for iNOS. **D.** A pleural metastatic adenocarcinoma showing positive cytoplasmic immunoreactivity for the monoclonal iNOS antibody in tumour cell. **E.** Another metastatic adenocarcinoma showing only weak positivity for the polyclonal iNOS anitbody. Positively stained reactive cells (arrowheads) can be seen among the neoplastic tumour cell islands.

Positivity was also observed in endothelial cells and in fibroblasts of the tumour stroma. There was no significant difference in the number of iNOS positive stromal macrophages and neutrophils between mesotheliomas and carcinomas. Furthermore, the number of positive stromal macrophages and neutrophils was not associated with the iNOS-positive mesotheliomas and/or carcinoma cases (data not shown).

Two iNOS antibodies were used to test the reproducibility of the iNOS immunostaining in the material. Generally, the polyclonal iNOS antibody gave a stronger immunoreaction compared with the monoclonal iNOS, whereas monoclonal iNOS antibody showed less background. There was a significant association between iNOS expression between the results obtained with the two iNOS antibodies ($P = 0.002$) in malignant mesotheliomas and in the whole material consisting also of the adenocarcinomas ($P < 0.001$). With both antibodies, there were significantly more

*© 2000 Cancer Research Campaign*

often iNOS negative mesotheliomas than metastatic adenocarcinomas ($P$ = 0.021 with the poly- and $P$ < 0.001 with the monoclonal antibody). Sarcomatoid mesotheliomas displayed significantly more often no immunostaining compared to epithelial or mixed mesotheliomas ($P$ = 0.001 with the poly- and $P$ = 0.023 with the monoclonal antibody). No association was found between iNOS immunoreactivity and patient survival ($P$ = 0.30 and $P$ = 0.16 for the poly- and monoclonal antibody, by the log rank test). In order to test the reproducibility of the results, they were evaluated by another pathologist (PP) using the same scoring system. The association between the evaluations of the two observers was significant ($P$ < 0.0001, Fisher's exact test).

The average apoptotic index in mesotheliomas was 1.07% (range 0.0–4.8%) and the average number of vessels/HPF 8.7 (range 1–58). There was no statistically significant association between iNOS expression and apoptosis or vascular density in mesotheliomas ($P$ = 0.07 and $P$ = 0.29 for the monoclonal and $P$ = 0.95 and $P$ = 0.25 for the polyclonal antibody). No association was found between patient survival and iNOS immunoreactivity in malignant mesotheliomas ($P$ = 0.251 and $P$ = 0.170 for the poly- and monoclonal antibody, log rank).

In metastatic adenocarcinomas the mean apoptotic index was 1.73% (range 0.07–7.01%) and the average vascular density 6.88/HPF (range 1.38–20.40). There was no statistically significant difference in apoptosis or vascular density as compared with mesotheliomas ($P$ = 0.12 and $P$ = 0.33, respectively). There was no statistically significant association between iNOS expression and apoptosis or vascular density in metastatic adenocarcinomas ($P$ = 0.73 and $P$ = 0.37 for the monoclonal and $P$ = 0.17 and $P$ = 0.82 for the polyclonal andibody).

To further confirm the synthesis of iNOS in malignant mesothelioma cells iNOS mRNA and protein expression was assessed in four malignant mesothelioma cell lines and also in transformed mesothelial cells (Met5A) in culture. Expression of iNOS mRNA was found in all five cell lines studied (Figure 2). Similarly, immunohistochemical expression of iNOS for the polyclonal antibody could be found in all cell lines investigated. By showing iNOS mRNA synthesis and immunohistochemical iNOS expression in all these cell lines these additional experiments confirmed the finding that neoplastic mesothelial cells are capable of synthesizing iNOS also in vitro.

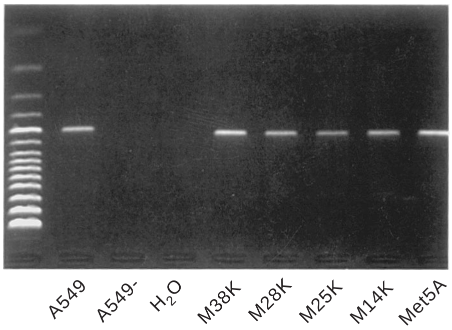

**Figure 2** The RT-PCR experiment for the mesothelioma cell lines. Lane 1: The positive control (A549 lung carcinoma cell line). Lane 2: Negative control, reverse transcriptase omitted. Lane 3: Negative control, substituting the RNA sample with water. Lanes 4–8: M38K, M28K, M25K and M14K mesothelioma cell lines and Met5A mesothelial cells. All these cell lines as well as A549 positive control cell line show a clear 500 bp band for iNOS mRNA.

### DISCUSSION

This study shows that the majority of malignant mesotheliomas express strong iNOS immunoreactivity. In contrast, its expression is infrequently found in non-neoplastic healthy mesothelium. When comparing iNOS reactivity in histologically different subtypes of mesothelioma, epithelial and biphasic subtypes expressed significantly more often iNOS positivity than the sarcomatoid subtype suggesting that its expression is especially a trait of the epithelial subtype.

The pathogenesis of mesothelioma is associated with asbestos fibres but a remarkable part of mesotheliomas (15%) can develop without a previous exposure to asbestos fibres (Jaurand, 1997). The present study with adenocarcinomas metastasized to pleura showed at least the same intensity of iNOS as was found in pleural mesotheliomas. Thus other factors than asbestos fibres probably cause the induction of iNOS in mesothelioma. Most likely these factors include increased levels of cytokines and growth factors which are produced by a variety of cells present in these conditions. Interestingly, our cell culture studied showed that malignant mesothelioma cells are able to synthesize iNOS also in vitro. This indirectly indicates that iNOS expression in malignant mesothelioma could be autocrinically regulated. In fact, mesotheliomas have been shown to be able to synthesize some interleukins and growth factors such as IL-6, IL-8 and to a lesser extent IL-1 and TNF$\alpha$ (Monti et al, 1994; Galffy et al, 1999).

Increased expression of iNOS has been found both in the tumour cells and in adjacent nonmalignant cells such as reactive macrophages and endothelial cells of the tumour tissue (Thomsen et al, 1994; Thomsen et al, 1995; Ambs et al 1998a; Ambs et al, 1998b; Klotz et al, 1998). Our results agree with these findings and show that both malignant mesothelioma and adenocarcinoma tumour cells as well as non-malignant stromal cells of both tumours are capable of iNOS synthesis. Most metastatic adenocarcinomas consisted of cases originating from breast and lung. A high frequency of iNOS expression in them is paradoxical since iNOS expression has been suggested to inhibit the metastatic potential of tumour cells (Xie and Fidler, 1998). On the other hand our results are consistent with recent experimental studies on iNOS in rat mesothelium (Choe et al, 1998; Tanaka et al, 1998) and suggest that the secretion of iNOS inducing cytokines may be higher in pleural tissues than in other metastatic sites.

Strong expression of iNOS in malignant pleural tumours suggests that NO synthesis may play an important role in the development and growth of these malignancies. Apoptosis has an important role in carcinogenesis and tumour progression, and it is known that NO may be either pro- or anti-apoptotic depending on the cell type, NO concentration and/or experimental conditions (Shen et al, 1998). NO also leads to the accumulation of wild type p53 and bax resulting in increased apoptosis (Ambs et al, 1998a). Accumulation of wild type p53, on the other hand, leads to inhibition of iNOS synthesis; thus there is a negative feedback loop between NO and iNOS synthesis (Ambs et al, 1998a). Also the family of bcl-2 proteins regulates or modulates apoptosis caused by NO. Bcl-2, for example, inhibits NO induced apoptosis (Kim et al, 1998). In mesotheliomas and mesothelial cell lines bcl-2 expression is infrequent while bax expression is frequently found (Segers et al, 1994; Narasimhan et al, 1998; Soini et al, 1999). NO has also been reported to inhibit APO-1/FAS mediated apoptosis (Mannick et al, 1997; Dimmeler et al, 1998). In our study we could

 © 2000 Cancer Research Campaign not find any significant association between apoptosis and iNOS expression. This may reflect the dual influence of NO on apoptosis and the complex regulation of apoptosis by other factors such as the bcl-2 family proteins.

Expression of iNOS also influences tumour vascularity by upregulating the synthesis of vascular endothelial growth factor (VEGF) thus promoting angiogenesis in tumour tissue (Ziche et al, 1997). Inducible NOS expression in mesothelial tumours might thus serve as a factor promoting tumour growth through stimulation of angiogenesis. In our material of malignant mesotheliomas there was no significant association between iNOS expression and vascular density suggesting that iNOS synthesis by mesothelioma tumour cells does not play a significant role in angiogenesis. Regulation of angiogenesis is, however, complex and confounded by various factors, such as the presence of iNOS-synthesizing stromal cells. In our pleural samples of malignant mesothelioma there were many reactive macrophages, neutrophils, fibroblasts and endothelial cells which were shown to express iNOS strongly. Surely, their presence would further modulate the internal milieu of the tumours and influence both angiogenesis and apoptosis in malignant pleural tumours.

In conclusion, our results show prominent expression of iNOS in malignant mesothelioma and metastatic adenocarcinoma of the pleura when compared to healthy pleural mesothelium. Also in vitro malignant mesothelial cell lines could be shown to express iNOS. This finding suggests that NO synthesis modulates the growth and progression of these tumours. Contrary to some previous notions, metastatic adenocarcinomas also expressed iNOS to a considerable degree. The total effect of iNOS on tumour behaviour in malignant mesotheliomas is probably a complex phenomenon where also stromal cells play an important role.

### ACKNOWLEDGEMENTS

The expert technical assistance of Mr Manu Tuovinen and Mrs Satu Koljonen is greatly appreciated. The cell lines were kindly provided by Dr Kaija Linnainmaa, Occupational Health Institute, Helsinki, Finland. This study was supported by the Finnish Cancer Societies and by the Finnish Anti-Tuberculosis Association Foundation.

### REFERENCES

Ambs S, Hussain P and Harris CC (1997) Interactive effects of nitric oxide and the p53 tumor suppressor gene in carcinogenesis and tumor progression. *FASEB J* **11**: 443–448

Ambs S, Bennett WP, Merriam WG, Ogunfusika MO, Oser SM, Khan MA, Jones RT and Harris CC (1998) Vascular endothelial growth factor and nitric oxide synthase expression in human lung cancer and the relation to p53. *Br J Cancer* **78**: 233–239

Ambs S, Merriam WG, Bennett WP, Felley-Bosco E, Ogunfusika MO, Oser SM, Klein S, Shields PG, Billiar TR and Harris CC (1998) Frequent nitric oxide synthase-2 expression in human colon adenomas: implication for tumor angiogenesis and colon cancer progression. *Cancer Res* **58**: 334–341

Battifora H and McCaughet WTE (1994) Tumors of the serosal memranes. Atlas of Tumor Pathology, Third Series, Fascicle 15. Armed Forces Institute of Pathology, Washington, DC, 15–88

Beckman JS and Koppenol WH (1996) Nitric oxide, superoxide, and peroxynitrite: the good, the bad, and the ugly. *Am J Physiol* **271**: C1424–C1437

Belvisi M, Barnes PJ, Larkin S, Yacoub M, Tadjkarimi S, Williams TJ and Mitchell JA (1995) Nitric oxide synthase activity is elevated in inflammatory lung disease in humans. *Eur J Pharm* **283**: 255–258

Chao CC, Park SH and Aust AE (1996) Participation of nitric oxide and iron in the oxidation of DNA in asbestos-treated human lung epithelial cells. *Arch Biochem Biophys* **326**: 152–157

Chen Q, Marsh J, Ames B and Mossman BT (1996) Detection of 8-oxo-2′deoxyguanosine, a marker of oxidative DNA damage, in culture medium from human mesothelial cells exposed to crocidolite asbestos. *Carcinogenesis* **17**: 2525–2527

Choe N, Tanaka S and Kagan E (1998) Asbestos fibers and interleukin-1 upregulate the formation of reactive nitrogen species in rat pleural mesothelial cells. *Am J Respir Cell Mol Biol* **19**: 226–236

Dimmeler S, Haendeler J, Sause A and Zeiher AM (1998) Nitric oxide inhibits APO-1/Fas-mediated cell death. *Cell Growth Differ* **9**: 415–422

Galffy G, Mohammed KA, Dowling PA, Nasreen N, Ward MJ and Antony VB (1999) Interleukin 8: an autocrine growth factor for malignant mesothelioma. *Cancer Res* **59**: 367–371

Geller DA, Lowenstein CS, Shapiro RA, Nussler AK, Di Silvio M, Wang SC, Nakayama DK, Simmons RL, Snyder SH and Billiar TR (1993) Molecular cloning and expression of inducible nitric oxide synthase from human hepatocytes. *Proc Natl Acad Sci USA* **90**: 3491–3495

Jaurand MC (1997) Mechanisms of fiber-induced genotoxicity. *Environ Health Perspectives* **105**: 1073–1084

Kamp DW and Weitzman SA (1999) The molecular basis of asbestos induced lung injury. *Thorax* **54**: 638–652

Kim YM, Kim TH, Seol TW, Talanian RV and Billiar TR (1998) Nitric oxide suppression of apoptosis occurs in association with an inhibition of bcl-2 cleavage and cytochrome c release. *J Biol Chem* **273**: 31437–31441

Kinnula VL (1999) Oxidant and antioxidant mechanisms of asbestos fibers in the lung and malignant lung diseases. *Eur Resp J* **14**: 706–716

Klotz T, Bloch W, Volberg C, Engelmann U and Addicks K (1998) Selective expression of inducible nitric oxide synthase in human prostate carcinoma. *Cancer* **82**: 1897–1903

Kobzik L, Bredt DS, Lowenstein CJ, Drazen J, Gaston B, Sugarbaker D and Stamler JS (1993) Nitric oxide synthase in human and rat lung: immunocytochemical and histochemical localization. *Am J Respir Cell Mol Biol* **9**: 371–377

Li JT (1997) Mechanisms of asthma. *Curr Opin Pulm Med* **3**: 10–16

Mannick JB, Miao XQ and Stamler JS (1997) Nitric oxide inhibits Fas-induced apoptosis. *J Biol Chem* **272**: 24125–24128

Moilanen E, Moilanen T, Knowles R, Charles I, Kadoya Y, al Saffar N, Revell PA and Moncada S (1997) Nitric oxide synthase is expressed in human macrophages during foreign body inflammation. *Am J Pathol* **150**: 881–887

Monti G, Jaurand MC, Monnet I, Chretien P, Saint-etienne L, Zeng L, Portier A, Devillier P, Galanaud P and Bignon J (1994) Intrapleural production of interleukin 6 during mesothelioma and its modulation by gamma-interferon treatment. *Cancer Res* **54**: 4419–4423

Mossman BT, Kamp DW and Weitzman SA (1996) Mechanisms of carcinogenesis and clinical features of asbestos-associated cancers. *Cancer Invest* **14**: 466–480

Narasimhan SR, Yang L, Gerwin BI and Broaddus VC (1998) Resistance of pleural mesothelioma cell lines to apoptosis: relation to expression of bcl-2 and bax. *Am J Physiol (Lung Cell Mol Physiol)* **275**: L165–L171

Pelin-Enlund K, Husgafvel-Pursiainen K, Tammilehto L, Klockars M, Jantunen K, Gerwin BI, Harris CC, Tuomi T, Vanhala E, Mattson K and Linnainmaa K (1990) Asbestos-related malignant mesothelioma: growth, cytology, tumorigenicity and consistent chromosome findings in cell lines from five patients. *Carcinogenesis* **11**: 673–681

Quinlan TR, Berube KA, Hacker MP, Taatjes DJ, Timblin CR, Goldberg J, Kimberly P, O'Shaughnessy P, Hemenway D, Torino J, Jimenez LA and Mossman BT (1998) Mechanisms of asbestos-induced nitric oxide production by rat alveolar macrophages in inhalation and in vitro models. *Free Rad Biol Med* **24**: 778–788

Saleh D, Barnes PJ and Giaid A (1997) Increased production of the potent oxidant peroxynitrite in the lungs of patients with idiopathic pulmonary fibrosis. *Am J Respir Crit Care Med* **155**: 1763–1769

Segers K, Ramael M, Singh SK, Weyler J, Van Meerbeeck J, Vermeire P and Van Marck E (1994) Immunoreactivity for bcl-2 protein in malignant mesothelioma and non-neoplastic mesothelium. *Virchows Arch* **424**: 631–634

Shen YH, Wang XL and Wilcken DE (1998) Nitric oxide induces and inhibits apoptosis through different pathways. *FEBS Lett* **14**: 125–131

Soini Y, Kinnula V, Kaarteenaho-Wiik R, Kurttila E, Linnainmaa K and Pääkkö P (1999) Apoptosis and expression of apoptosis influencing proteins bcl-2, bax, mcl-1 and bcl-x in malignant mesothelioma. *Clin Cancer Res* **5**: 3508–3515

Tanaka S, Choe N, Hemenway DR, Zhu S, Matalon S and Kagan E (1998) Asbestos inhalation induces reactive nitrogen species and nitrotyrosine formation in the lungs and pleura of the rat. *J Clin Invest* **102**: 445–454

Thomas G, Ando T, Verma K and Kagan E (1994) Asbestos fibres and interferon-gamma upregulate nitric oxide production in rat alveolar macrophages. *Am J Respir Cell Mol Biol* **11**: 707–715

© 2000 Cancer Research Campaign 

Thomsen LL, Lawton FG, Knowles RG, Beesley JE, Riveros-Moreno V and Moncada S (1994) Nitric oxide synthase activity in human gynecological cancer. *Cancer Res* **54**: 1352–1354
Thomsen LL, Miles DW, Happerfield L, Bobrow LG, Knowles RG and Moncada S (1995) Nitric oxide synthase activity in human breast cancer. *Br J Cancer* **72**: 41–44
Walz R and Koch HK (1990) Malignant pleural mesothelioma: some aspects of epidemiology, differential diagnosis, and prognosis: histological and immunohistochemical evaluation and follow-up of mesotheliomas diagnosed from 1964 to January 1985. *Pathol Res Pract* **186**: 124–134
Wink DA and Mitchell JB (1998) Chemical biology of nitric oxide: insights into regulatory, cytotoxic, and cytoprotective mechanisms of nitric oxide. *Free Rad Biol Med* **25**: 434–456
Xie K and Fidler IJ (1998) Therapy of cancer metastasis by activation of the inducible nitric oxide synthase. *Cancer Met Rev* **17**: 55–75
Zhao H, Dugas N, Mathiot C, Delmer A, Dugas B, Sigaux F and Kolb J-P (1998) B-cell chronic lymphocytic leukemia cells express a functional inducible nitric oxide synthase displaying anti-apoptotic activity. *Blood* **92**: 1031–1043
Ziche M, Morbidelli L, Choudhuri R, Zhang H-T, Donnini S, Granger HJ and Bicknell R (1997) Nitric oxide synthase lies downstream from vascular endothelial growth factor-induced but not basic fibroblastic growth factor-induced angiogenesis. *J Clin Invest* **99**: 2625–2634

 *© 2000 Cancer Research Campaign*
